# Lessons Learnt from Assembling Screening Libraries for Drug Discovery for Neglected Diseases

**DOI:** 10.1002/cmdc.200700139

**Published:** 2007-12-06

**Authors:** Ruth Brenk, Alessandro Schipani, Daniel James, Agata Krasowski, Ian Hugh Gilbert, Julie Frearson, Paul Graham Wyatt

**Affiliations:** [a] University of Dundee, College of Life Sciences, James Black CentreDow Street, Dundee DD1 5EH, UK, Fax: (+44)1382-386373

**Keywords:** compound selection, high-throughput screening, kinase inhibitors, neglected diseases, virtual screening

## Abstract

To enable the establishment of a drug discovery operation for neglected diseases, out of 2.3 million commercially available compounds 222 552 compounds were selected for an in silico library, 57 438 for a diverse general screening library, and 1 697 compounds for a focused kinase set. Compiling these libraries required a robust strategy for compound selection. Rules for unwanted groups were defined and selection criteria to enrich for lead-like compounds which facilitate straightforward structure–activity relationship exploration were established. Further, a literature and patent review was undertaken to extract key recognition elements of kinase inhibitors (“core fragments”) to assemble a focused library for hit discovery for kinases. Computational and experimental characterisation of the general screening library revealed that the selected compounds 1) span a broad range of lead-like space, 2) show a high degree of structural integrity and purity, and 3) demonstrate appropriate solubility for the purposes of biochemical screening. The implications of this study for compound selection, especially in an academic environment with limited resources, are considered.

## Introduction

The pharmaceutical industry has a long track record of delivering innovative therapeutics. However, because of economic reasons, this industry is mainly investing in therapeutic areas for which a commercially viable return can be expected.^1^ Consequentially, only little translational research is carried out on orphan or neglected diseases where either few or poor people are affected.^2^,^3^ For instance, of the more than 1300 new drugs introduced between 1975 and 1999, only 13 were for infectious parasitic diseases, which together account for a third of the worldwide disease burden.^4^ Therefore, to tackle orphan or neglected diseases either the pharmaceutical industry must be given incentives to work on them or not-for-profit organisations need to be enabled to do drug discovery.^3^,^5^ Increasingly, academic groups are either partnering with industry or are recruiting experienced people from industry to guide and support their drug discovery efforts, creating an environment which allows professional translational research and opens up access to key technologies, such as screening facilities and libraries for hit discovery.^6^

Over recent decades library screening has become an important source of hits for drug discovery programmes.^7^,^8^ Three main technologies are used: 1) virtual screening of in silico libraries to identify small sets of compounds for biochemical assays,^9^,^10^ 2) fragment-based screening using high-throughput x-ray crystallography or NMR methods to discover relatively small compounds which bind with high ligand efficiency to the target,^11^ and 3) high-throughput biochemical screening (HTS) of either diverse chemical libraries or focused libraries tailored for specific gene families.^12^,^13^ After initially very disappointing HTS outcomes it became evident that the quality of the compound collection is crucial for success.^12^,^14^ It is now generally accepted that the physicochemical properties of the compounds, the absence of compounds containing toxic or reactive moieties, and lead- or drug likeness are important factors when compiling screening libraries.^12^,^15^ A more controversial point is the required size and diversity of general purpose screening libraries. In the pharmaceutical industry, these libraries can contain over a million compounds; usually a combination of commercially available and proprietary compounds.^8^ These organisations balance the cost of screening many hundreds of thousands of inactive compounds against the benefits such as directly establishing structure–activity relationships (SAR) from the screening data and increasing the chances of identifying a broad range of hit series. The latter point is particularly important in a competitive environment to maximise the chances of discovering structural classes without patent issues.

On the other hand, smaller libraries containing between 14000 and 135000 compounds have been successfully screened for hit discovery.^16^–^19^ In a less competitive area with restricted resources, such as translational research for orphan or neglected diseases, these smaller libraries are very appealing. Such collections might not reveal every single hit series that would have been discovered using larger libraries and only limited initial SAR, but by careful selection of libraries to cover lead-like chemical space in as diverse as possible manner, they should generate sufficient information to initiate a drug discovery programme. In this case the initial screen will need to be followed by further rounds of purchase of compound analogues and screening to validate and expand the SAR around the hits.

At the University of Dundee, we are particularly interested in drug discovery for neglected parasitic diseases. A diverse set of enzymes targets in various pathways has been validated as potential drug targets for these diseases.^20^ Many of these targets are novel with little precedence for drug discovery and either no or only limited drug-like inhibitors are known. To tackle these targets efficiently with up-to-date drug discovery methods, a diverse screening library is needed.^6^,^12^ The three dimensional structures for some targets have been determined allowing virtual screening as a complementary approach to high volume biochemical screening.^21^ In addition, there are also potential parasite targets that are homologues of well-known drug targets, such as parasite protein kinases.^22^ Extensive research, especially in the oncology area, has resulted in approved drugs and demonstrated that kinases are highly druggable.^23^ Many chemical scaffolds that inhibit human kinases are known and it can be assumed that these scaffolds can also serve as starting points for parasitic kinase inhibitors accelerating the entry into kinase drug discovery for parasitic indications.^6^

Due to the large attrition rate in drug discovery^6^,^24^ and the diversity and lack of precedent for many potential parasite drug targets,^20^ we decided not to focus our drug discovery efforts on one particular target class but to cover a broad range of targets. To enable this concept we compiled three different libraries: one diverse in silico library for virtual screening, one diverse screening compound library, and a focused compound library for the discovery of kinase inhibitors. Herein, we report on our approach to assemble these libraries and on their quality assessment. Furthermore, we also discuss general lessons which can be drawn for compound selection.

## Overview of the compound selection procedure

Three different libraries were assembled: a virtual screening set representing valuable starting points for lead optimisation programmes, a HTS library intended to be a diverse subset of these compounds, and a kinase library containing compounds that are likely to inhibit kinases. To ensure selection of high quality compounds, the following selection criteria were defined (Table 1):

**Table 1:** Compound selection criteria.

Selection criteria	Definition
Absence of unwanted functionalities:	No unwanted groups (see supplementary material)
Lead-like properties:	10–27 heavy atoms
	<4 hydrogen-bond donors
	<7 hydrogen-bond acceptors
	0 <(hydrogen-bond donors + hydrogen bond acceptors)<10
	0–4 Clog*P*/Clog*D*
	If the compound contains only one ring system at least one atom has to be outside the ring.
Limited complexity:	<8 rotatable bonds
	<5 ring systems
	No ring systems with more than two fused rings

**Absence of unwanted functionalities**. Compounds containing unwanted functionalities were removed as it is not desirable to waste resources removing such functionalities in the hit optimization phase. These included potentially mutagenic groups such as nitro groups, groups likely to have unfavourable pharmacokinetic properties such as sulfates and phosphates; and reactive groups such as 2-halopyridines or thiols (Table S1). Furthermore, compounds which are likely to interfere with typical HTS assays were also excluded.

**Lead-like properties**. Generally, molecular weight, lipophilicity, and number of hydrogen-bond donors and acceptors of a compound are increased in the lead optimisation process.^25^,^26^ Consequently, we decided not to purchase drug-like compounds as defined by “Lipinski’s rule of 5” but to select compounds that are smaller and less hydrophobic to leave opportunities for optimisation.^27^ In particular, we restricted the Clog*P*/Clog*D* to between zero and four, the number of hydrogen-bond donors and acceptors to fewer than four and seven, respectively, and the number of heavy atoms to between ten and 27.

**Limited complexity**. Given the low probability of any one chemical hit (series) being successfully progressed to a preclinical candidate, we sought chemically tractable compound scaffolds to allow the facile synthesis of diverse arrays of compounds to explore structure–activity relationships (SAR), allowing rapid go/no go decisions on any particular series. Therefore, only compounds with limited complexity defined as fewer than eight rotatable bonds, fewer than five ring systems, and no ring systems with more than two fused rings were included.

**Figure 1:**
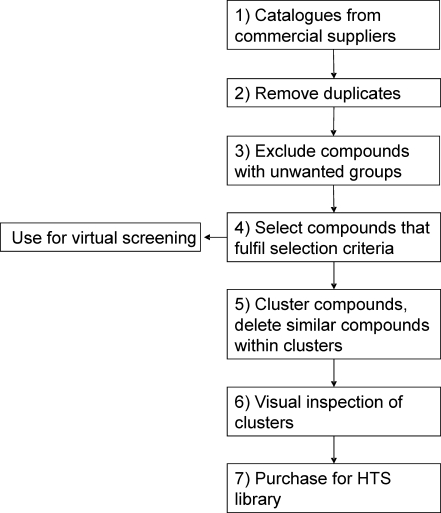
Workflow for compound selection.

**figure 2:**
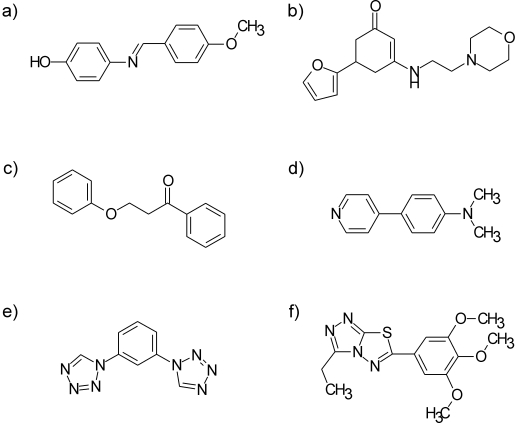
Examples of rejected compounds after visual inspection. a) and b) contain potentially reactive groups, c) and d) are under functionalised and e) and f) are over functionalised.

A hierarchical filter protocol was established to enrich the desired compounds (Figure 1). After pooling supplier catalogues and filtering for duplicates, compounds that contained unwanted functionalities were removed. Definitions of these groups were derived from the literature, and augmented with our own in-house rules based on medicinal chemical experience (Table S1).^28^,^29^ In the next step we filtered for compounds with lead-like properties and limited complexity (Table 1). All compounds passing these filters were regarded as, in general, valuable starting points for medicinal chemistry programmes and are used for virtual screening campaigns (VS set). Finally, for the HTS library the number of compounds was reduced further by cluster analyses and visual inspection. All compounds in the VS set were clustered based on Tanimoto similarity. Compounds within a cluster with a pairwise Tanimoto similarity >0.9 to a member of the same cluster were rejected to avoid redundant information. In the last step at least one representative of each cluster was visually inspected to remove compounds that, based on our experience, are unsuitable starting points for chemistry programs because they: 1) contain potentially reactive or toxic groups for which no filter rules were defined (Figure 2a,b); 2) appear under functionalised compared to their size (Figure 2c,d); or 3) are already highly functionalised and therefore left limited options for optimisation (Figure 2e,f). This last visual inspection was carried out by two people, to provide a consensus and to ensure consistency.

**Figure 3:**
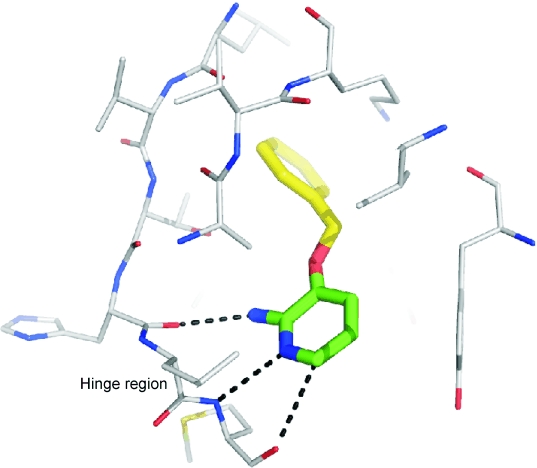
Typical binding mode of a kinase inhibitor in the adenine pocket (PDB code 1w7h). The core fragment (green carbon atoms) forms hydrogen bonds with the backbone amide groups of the hinge region; the substituent (yellow carbon atoms) addresses a further pocket.

For the assembly of the focused kinase library a more rational approach was chosen. Numerous kinase inhibitors belonging to different chemical classes have been described.^23^ Most of these inhibitors contain a core fragment that binds in the kinase adenine binding pocket and forms hydrogen-bonds with backbone amide groups of the amino acids that comprise the so-called hinge region (Figure 3).^30^ Specificity for different kinases is achieved by appropriate decoration of these core fragments with groups that enable interactions with the more variable parts of adjacent binding pockets. A focused library with a relatively high hit rate for a diverse panel of kinases should therefore contain a broad variety of core fragments that are decorated with diverse substituents. Following these considerations, again a hierarchical filter protocol was established. In the first step, an extensive literature and patent review was carried out to assemble a list of kinase inhibitors with core fragments that potentially bind into the adenine pockets of kinases. In the next step, the VS set was screened for compounds that contained the desired core fragments. In the last step, where more than 50 examples of a fragment were retrieved, in iterative cycles similar representatives of the same core fragment were rejected until 50 compounds were left.

## Methods

### Descriptor calculations

In-house Python scripts based on Openeye’s OEToolkit (Openeye, Santa Fe, USA) were used for compound manipulation and calculating descriptors for the number of heavy atoms, hydrogen-bond donors and acceptors, ring systems, and rotatable bonds. SD files provided by the suppliers were converted into SMILES strings. Protonation and tautomeric states of the compounds were standardised based on predefined substructure patterns to remove duplicates. In silico ADME parameters were calculated using ADMEnsa Interactive (BioFocus DPI, Saffron Walden, UK). Sybyl (Tripos, St. Louis, USA) was used to calculate Clog*P* values. Compounds containing groups that are charged at physiological pH were neutralised before calculating Clog*P* values. The Clog*P* values obtained for compounds where converted to clog*D* values by equation 1 and a p*K*_a_ of four was assumed for acetyl-sulfonamides, a p*K*_a_ of five for carboxylic acids and tetrazoles, a p*K*_a_ of six for aromatic thiols, and a p*K*_a_ of nine for amines.

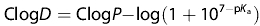



Compounds and their descriptors were stored in a MySQL database and visualised using Vida and the Ogham package (Openeye).

### Compound clustering

Compounds were clustered using the Jarvis-Patrick algorithm of the Daylight clustering package (Daylight, Aliso Viejo, USA). Compounds had to have at least nine of 15 neighbours in common to be allowed to belong to the same cluster and compounds for which the nearest neighbour had a Tanimoto coefficient of less than 0.8 were classified as singletons. Compounds within a cluster that had a Tanimoto coefficient of larger than 0.9 with a member of the same cluster were deleted. In the first round of purchasing we required a minimum cluster size of five. In the second purchase round no minimum cluster size was required.

### Kinase library

Kinase core fragments were manually extracted from literature and patents and represented by SMARTS strings with the help of Openeye’s mdl2sma tool. Python scripts using the Openeye’s OEToolkit were used to retrieve matching compounds. In cases in which a core fragment occurred in more than 50 different compounds, the cluster size was reduced in iterative cycles. In the first cycle, all compounds that had a Tanimoto similarity >0.9 (based on Daylight fingerprints) to a member in the same cluster were removed. In subsequent cycles, the Tanimoto threshold was reduced stepwise by 0.1 until the desired cluster size was reached.

### Automatic core fragment extraction

**Figure 4:**
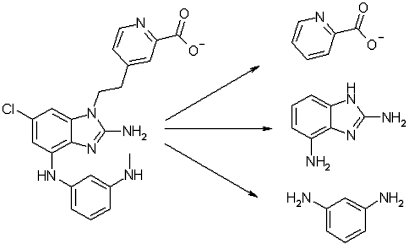
An example of core fragment extraction. All halogen atoms and carbon atoms that are not part of a polar functional group or a ring system are removed. Functional groups linking ring systems are added to all adjacent rings.

In a variation of previously published methods, a core fragment was defined as a ring plus the directly attached polar functional groups (Figure 4).^31^,^32^ Polar functional groups were specified as nitrogen, oxygen, or sulfur atoms directly linked to these types of atoms, carbonyl groups, or double or triple bonded carbon atoms. Fragments only containing carbon atoms or carbon atoms and aromatic sulfur or oxygen atoms were discounted. The details of the method will be published elsewhere.

### Solubility screening

The complete compound collection was solubilised as 10 mm master stocks in 100% DMSO and daughter sets were prepared at 3 mm for routine screening campaigns. All sets were stored at −20°C under reduced humidity conditions prior to use. For the screen, 1 μL of each compound was dry spotted into 384 well plates (Hummingbird, Genomic Solutions Inc.) prior to addition of 100 μL phosphate buffered saline (PBS), pH 7.4 (Flexdrop, Perkin–Elmer Inc.), resulting in a compound concentration per well of 30 μm at 1% DMSO. After equilibration for 1 h at room temperature plates were read at A_620nm_ in an Envision plate reader (Perkin–Elmer Inc.). Pyrene (30 μm) was used as an internal plate control (16 wells per plate); background equated to PBS alone (16 wells per plate).

## Results

### Compound selection for the VS and HTS sets

**Figure 5:**
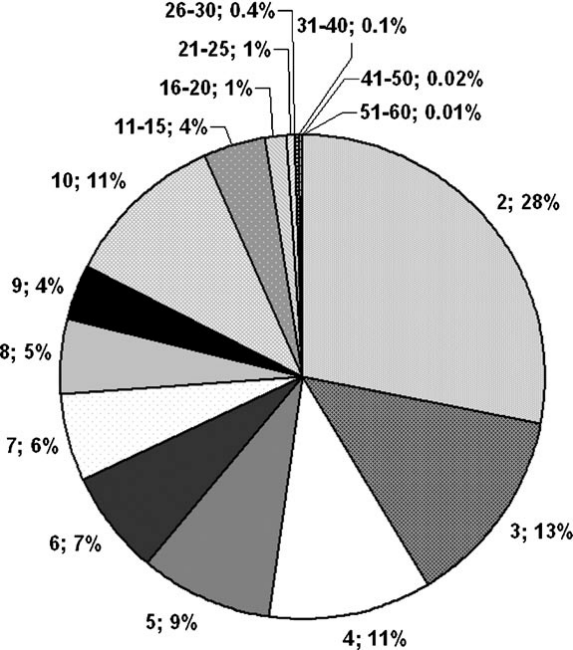
Distribution of cluster sizes in filtered library after similar compounds within clusters were removed. The slices indicate how often a particular cluster size occurs, for example, “2; 28%” means that 28% of the total number of clusters have a cluster size of two.

For logistical reasons compound selection for the VS and HTS sets was performed in two rounds. In the first round, only one supplier offering a large number of compounds (Chemdiv) was considered. In the second round we then increased the size of the library by adding compounds from additional suppliers (Table 2). In total, 605230 compounds were included in the first round and 2262339 in the second round (Table 3). Removal of duplicates and compounds with unwanted functionalities resulted in 486453 and 932081 compounds, respectively. Out of those, 95469 and 222552 compounds, respectively fulfilled the criteria for lead-like properties (Table 1) and were included in the VS set. For the HTS set the number of compounds was reduced further by cluster analyses and visual inspection. Clustering the VS set of the first purchasing round in which a minimum cluster size of five was required resulted in 3747 clusters and the second round, in which no cut-off was applied, resulted in 9705 clusters and 31105 singletons (Figure 5). The maximum cluster size was 62 and 60 compounds, and 74.3% and 93.3% of the clusters contained ten or less members in the first and second round, respectively. Visual inspection of these clusters reduced the size further to 29206 and 55184 compounds, respectively, out of which 24047 compounds were purchased in the first purchasing round and 33391 in the second round (the large discrepancy between the numbers of compounds passing the visual inspection filter step and the number of compounds actually purchased in the second purchasing round is due to the fact that most of these compounds were already purchased previously).

**Table 2:** Suppliers considered.

Supplier	Number of compounds
Asinex	362493
Biofocus	17242
Bionet	44562
Chembridge	484141
Chemdiv	605230
IBS	377684
Maybridge	58855
Peakdale	8548
Sigma–Aldrich^[a]^	104421
Specs	195129
Tripos[a]	4064
Σ	2262339

[a] These suppliers provided prefiltered sets.

**Table 3:** Results of hierarchical compound filtering.[a]

Filter step	1^st^ Round	2^nd^ Round
1) available compounds	605230	2262339
2) removal of duplicates	601669	1752298
3) removal of compounds with unwanted functionalities	486453	932081
4) lead-like properties and limited complexity	95469	222552
5) Clustering	33702	89245
6) Visual inspection	29206	55184
7) Purchased	24047	33391

[a] In the first round only compounds from Chemdiv were considered, in the second round all available compounds from the suppliers listed in Table 1 were filtered.

### Compound selection for the focused kinase library

A literature and patent review identified 113 core fragments that potentially bind into the adenine pockets of kinases (Table 4, S2). Substructure searching of these core fragments in the VS set resulted in zero hits for 31 core fragments, less than 50 hits for 40 core fragments, and 50 or more hits for 42 core fragments. After rejecting similar compounds representing core fragments for which more than 50 examples were retrieved, 1697 compounds were identified.

**Table 4:** Examples of kinase inhibitor core fragments and their occurrences in the unfiltered and filtered library.

Core Fragment ID	Core fragment	Smarts string	Number of hits	
			Unfiltered library	Filtered library
1		[n!H0,nX2]1:c2:n:[c!H0]:c:n:c:2:[n!H0,nX2]:c:1	0	0
2		[nX2]1:c:c2:c:c:c:[nX2]:c:2:c:c:1!@[#7!H0]	6	0
3		[cH]1nc(cs1)!@C(=O)[#7!H0]	9	5
4		[nX2,nH]1[nX2,nH]c(c[cH]1)!@C(=O)[#7!H0]	223	27
5		[nX2]1:[cR2](!@[N!H0]):[cR2](!@-C!@#N):[cR2]:[cR2]:[cR2]:1	1500	254
6		[cH]1[cR2][cH]n[cR2]([cR2]1)!@[N!H0]	13703	3403

### Computational characterisation of the HTS library

**Figure 6:**
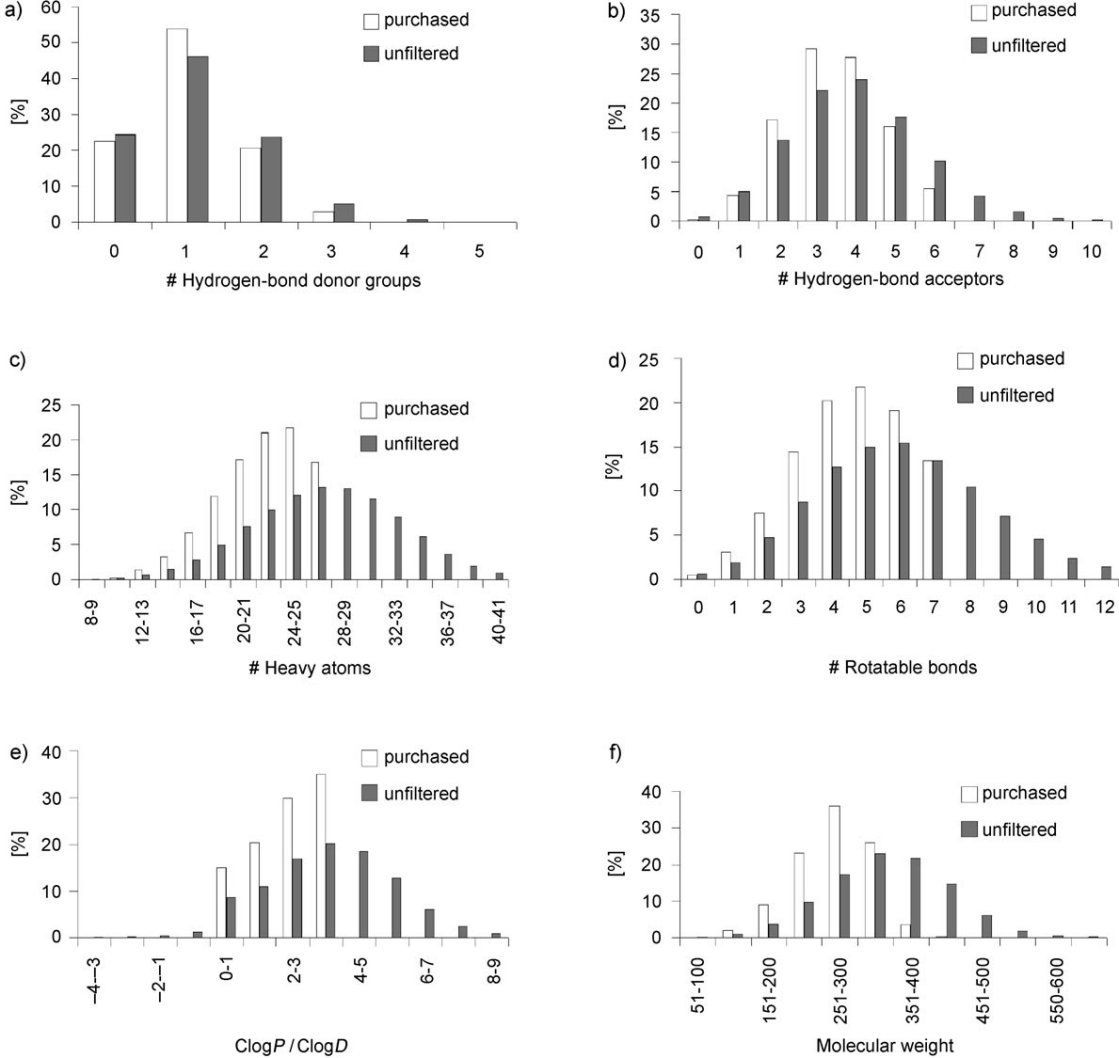
Frequency distribution histograms for a) number of hydrogen-bond donor groups, b) number of hydrogen-bond acceptors, c) number of heavy atoms, d) number of rotatable bonds, e) Clog*P* for neutral compounds and Clog*D* for compounds containing ionisable groups, and f) molecular weight in the purchased compound collection (white bars) and the unfiltered library (grey bars).

The content of the HTS library was characterised in terms of distribution of 1D properties and core fragment diversity. Frequency distribution histograms show that the selected compounds cover a broad range of lead-like chemical space (Figure 6). Most compounds in the purchased library have one hydrogen-bond donor group (54% of the library), 3–4 hydrogen-bond acceptors (57%), 20–25 heavy atoms (60%), 4–6 rotatable bonds (61%), and a Clog*P*/Clog*D* between three and four (35%). According to in silico ADME property calculations 74% of the compounds are able to penetrate the blood-brain barrier (log([brain/blood]) >−0.5), 96% are classified to be orally available (human intestinal absorption >30%), 79% inhibit the Ether-a-go-go-related gene (hERG) potassium channel only with pIC50 <6, 70% are not transported by P-glycoproteins (P-gp), and 30% show less than 80% plasma protein binding (Table 5).

**Table 5:** In silico ADME properties of the compounds in the purchased screening collection.

Property	Desired Value	% over threshold
Blood-brain barrier penetration (log([brain]:[blood]))	>−0.5	74
Human intestinal absorption	>30%	96
hERG pIC50	<6	79
Transport by P-glycoprotein	no	70
Plasma protein binding	<80%	30

**Figure 7:**
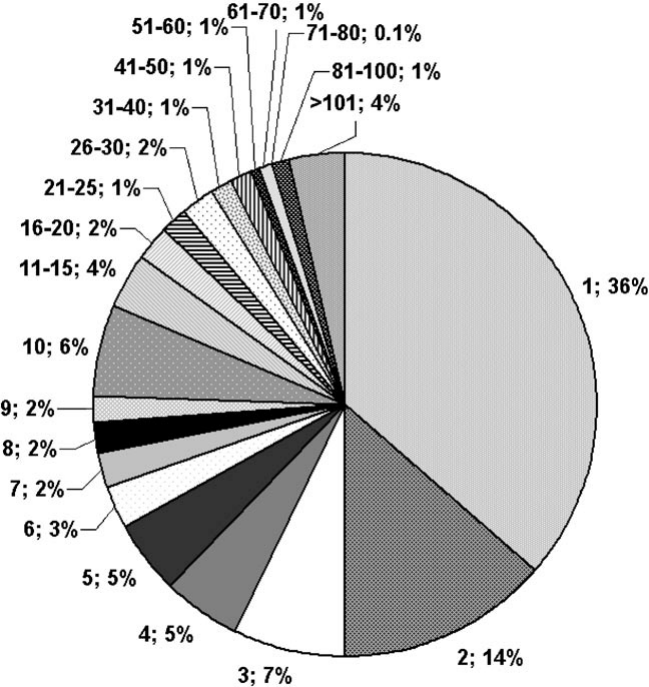
Core fragment representation in purchased library. The slices indicate the percentage of the total number of core fragments that are contained in a certain number of compounds, for example, “11–15; 4%” means that 4% of the core fragments are contained in 11–15 compounds.

To analyse the diversity of the library, core fragments were extracted from the compounds (Figure 4). The purchased library contains 4812 different core fragments. For 93% of these core fragments there are less than 50 different examples in the screening library and 50% of the fragments occur only in one or two compounds (Figure 7).

### Experimental characterisation of the HTS library

Experimental characterisation of the collection included assessment of purity and identity for a random sample and kinetic solubility of the entire collection. A randomly selected sample comprising 1% of the collection was checked for purity and structural integrity by LC-MS. The vast majority of the compounds (98%) gave the expected molecular identity according to molecular weight and 96% of those tested returned purities equal to or greater than our acceptance threshold of 90%. The majority of those which failed returned purities above 70%.

**Figure 8:**
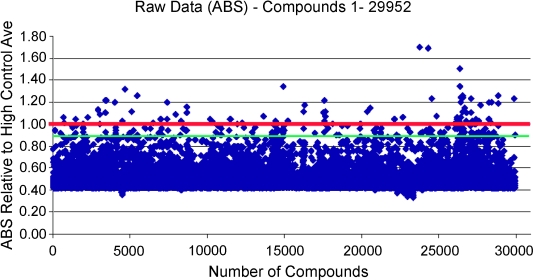
Distribution of solubility assessment readings for first 30,000 compounds at 30 μm. Test compound data are expressed relative to the positive control pyrene (solubility <10 μm) (red line). Green line denotes 3xSD below the mean pyrene control. All compounds with readings on or above the green line were annotated as having potential solubility issues at 30 μm.

The solubility screen demonstrated excellent statistical performance yielding mean A_620nm_ reading of 0.07±0.004 (mean ±SD) for the standard compound across a total of 178 assay plates, with a mean Z’ for the assay of 0.77±0.06. Figure 8 illustrates the distribution of compound readings for the first 30000 of the compounds tested, relative to the standard. Analysis of the complete data set revealed that 1.3% of the collection gave readings within 3 x SD and above the mean of the standard compound. We considered these compounds to have likely solubility issues at the typical concentration for primary screening (30 μm) and they were annotated in our database accordingly.

## Discussion

Virtual screening and large-scale biochemical screening are established tools for hit discovery.^10^,^12^ In the past, especially HTS has mainly taken place in industry but screening centres are increasingly being established in academia to address gaps left by industrial activities.^6^ As academic institutions often have no historical compound library, the opportunity arises to assemble a screening library from first principles. By going through two iterative cycles of compound selection we selected 57438 compounds for a general purpose screening library and 1697 compounds for a focused kinase set. Four points stand out from this exercise. First, by restricting compound selection to compounds that are diverse and lead-like, a HTS library of a size which is suitable for drug discovery in an academic setting can be assembled. Second, the selected compounds cover a broad range of lead-like chemical space and are generally of high quality in terms of purity and identity. Furthermore, triaging the commercially available compounds with our filter criteria led to a high rate of soluble compounds. Third, despite the size of the commercial compound space, compounds containing some rather simple core fragments are lacking. Fourth, the detailed implementation of a compound selection cascade is dependent upon prior drug discovery experience. We consider these points further below.

### Suitable screening sets for academic environments

Screening sets with more than one million compounds are often used in the pharmaceutical industry, but they are too costly and resource intensive for academic settings. However, even the large sets cover only a tiny fraction of drug-like chemical space. In addition, the hit rates with these large sets can still be very disappointing.^13^,^33^ By restricting compound size and complexity to those of lead-like compounds, smaller libraries which still provide a reasonable coverage of chemical space can be assembled, as the magnitude of chemical space is exponentially smaller for lead-like than for drug-like compounds.^34^ For instance, a theoretical study has shown that less than 14 million molecules consisting of up to eleven N, O, C, and S atoms can potentially be synthesised whereas drug-like chemical space was estimated to exceed 10^60^ molecules.^35^,^36^ Further, although the binding affinities of lead-like compounds can be weaker than those of drug-like compounds, smaller compounds have a higher likelihood of binding to a target.^25^ Finally, when going from lead to drug, compounds usually increase in size, lipophilicity, and number of hydrogen-bond donors and acceptors, again supporting the idea to start with molecules that are smaller than average drugs.^15^,^26^,^37^ But how small should the compounds be? At the extreme end are fragments, which are often described with the rule of three (MW<300, log*P* ≤3, number of hydrogen-bond donors ≤3, number of hydrogen-bond acceptors ≤6).^11^,^38^ Typical fragment libraries contain only between a few hundred and 10000 small compounds. Compared to conventional HTS hits the often less potent fragments have the advantage that they usually have an optimal fit to the binding site and can therefore readily be optimised. Biophysical methods such as NMR and X-ray crystallography are routinely used to identify these weak binding hits but Makara argues that when using more densely populated fragments sets, traditional screens can also be applied.^39^ Based on these considerations we decided to choose compounds that are at the upper end of the fragment space or slightly bigger and more complex (Table 1) with the goal of obtaining initial hits with affinities that are detectable in a biochemical assay setting but are still amenable to optimisation. After applying our selection criteria for unwanted groups, limited complexity, and lead-likeness, only a fraction of the commercially available compounds is left (Table 3). Out of 1.7 million examined compounds only 222552 (13%) pass all defined filters for compounds suitable for virtual screening. This set is already considerably smaller than typical screening libraries in industry. By rejecting similar compounds and visual inspection we reduced its size further to approximately 57000 compounds, an appropriately sized set for an academic setting.

### Quality of selected screening set

The selected compounds span a broad range of lead-like chemical space (Figure 6) and show overall good in silico ADME properties (Table 5). The high percentage of compounds that are predicted to penetrate the blood-brain barrier was not intended but is an added bonus as our primary disease focus, human African sleeping sickness, requires CNS active compounds to treat the late stage of the disease.^2^ Interestingly, the 1D properties are not equally represented by the compounds in the library but the representation follows an almost normal distribution for the number of hydrogen-bond donors, acceptors, heavy atoms, and rotatable bonds (Figure 6). The uneven distribution of 1D properties in the purchased library reflects the representation of these properties in the commercially available compounds. For the number of hydrogen-bond donors and acceptors the library of commercially available compounds peaks at similar values whereas 47% of the available compounds are larger than our chosen cut-off of 27 heavy atoms and 43% fall outside the required Clog*P*/Clog*D* range. Commercial libraries have clearly improved compared to the early days of combinatorial chemistry and HTS, but there is still room for improvement in the design of compounds for more resource efficient screening strategies.

The diversity of the screening library was assessed based on a representation of core fragments (Figure 4). The final set contains almost 5000 different fragments out of which 93% are represented by less than 50 different examples demonstrating that our compound selection and clustering protocol resulted in a diverse set (Figure 7). However, some core fragments are under-represented, for example, 50% of the core fragments occur only in one or two compounds. Therefore in the future, we will purchase additional compounds which either increase the representation of sparsely populated core fragments or contain new core fragments.

Structural integrity and purity analysis demonstrated that the compounds from a broad range of suppliers were of high quality, as almost all of the randomly tested compounds were the expected structure and at least 90% pure. Continuing LC-MS quality control of hits from screening campaigns gives a similar rate to those from the initial random sampling of 1% of the library.

A simple and rapid assessment of the solubility of all members of our new library revealed that only 1.3% of the compounds have solubility issues. As a result of the known performance problems of solubility prediction models across multiple compound series we did not attempt to calculate this property or use it as a selection filter.^40^ However, we hypothesised that selecting rather small, non highly lipophilic compounds for our library would be beneficial for aqueous solubility. This was indeed confirmed by the solubility screen.

### Gaps in commercial compound space

Assembling the focused kinase library where examples of specific structural classes were sought revealed gaps in commercial compound space. Almost 30% of the core fragments were not commercially available and 35% occurred in fewer than 50 compounds. This was not due to our filter criteria as these gaps also existed in the unfiltered library (Table 4). These results re-emphasise that it is not the absolute number of commercially available compounds that is critical but how diverse they are. Clearly, at least a third of the known kinase inhibitor cores could not be discovered with screening libraries assembled from commercially available compounds.

### Expert knowledge influences the detailed implementation of a compound selection cascade

The implementation of a compound selection cascade is biased by the experience and intuition of the medicinal chemists involved. It is generally accepted that compounds in a screening library should be diverse, lead-like or drug-like, and free of problematic functionalities. The differences arise when this concept is translated into a detailed protocol. How exactly are lead- and drug-likeness defined? Which moieties are considered problematic? Which diversity measure should be used? In industry, data generated in screens over the last decade can be mined to inform compound selection.^41^–^44^ Academia, however, is just starting to use this technology and does not have access to historical data. To avoid duplicating efforts and to enable research groups to learn from each other, it is desirable that the emerging screening centres share their compound selection protocols and screening results. In this context, PubChem is a promising start.

One lesson we learned from our selection process was how to ensure and assess the diversity of the library. During visual inspection of the clustering results, we noticed that: 1) some clusters contained compounds that appeared to have relatively little structural similarity, or 2) sometimes compounds that we identified as having structural similarity, were found in different clusters. Inspired by the core fragment-based approach used for assembling the focused kinase library we then tried to mimic that approach for the screening library. Interestingly, retrospective analysis showed that the applied clustering strategy resulted in a good representation of most of the core fragments (Figure 7) indicating that a broadly similar library would have been obtained if a core fragment-based approach for compound selection was adopted. However, as noted earlier, the extracted core fragments are more meaningful for medicinal chemists than a randomly chosen cluster representative during visual inspection.^31^,^32^,^45^ In addition, by using the core fragment-based approach, fewer singletons (1755 versus 31105) and clusters (3057 versus 9705) were found in the filtered library speeding up this process. For these reasons, we have already used this approach to extend the focused kinase set to 3885 compounds and are currently in the process of applying it to expand the general screening compound library.

## Conclusions

To select a library of a size that is suitable for an environment with restricted resources, such as hit discovery for orphan or neglected diseases, we established a series of selection filters to identify a lead-like library of compounds which would be regarded by medicinal chemists as good starting points for optimisation campaigns. Using this process we selected 222552 compounds for a virtual screening library and 57438 compounds for a general screening library. These compounds span a broad area of lead-like chemical space and show good in silico ADME properties. Chemical identity, purity, and solubility assessments and diversity analysis showed that the general screening library is of high quality. Furthermore, using a rational approach 1697 compounds were selected for a focused kinase set which was subsequently expanded to 3885 compounds.

Of course, the ultimate test of the quality of a chemical library is its ability to produce valuable chemical starting points in screening campaigns. Such campaigns are underway and preliminary results look promising. In the first HTS screen undertaken, 1.3% of compounds were found to be hits (as defined by showing >50% inhibition at 30 μm). These were reconfirmed and a further selection made for IC_50_ determination. This led to identification of a number of chemically tractable, lead-like compound series which were subsequently validated in terms of resynthesis, exploration of SAR, and mechanism of action studies. Furthermore, screening the kinase set against a parasitic kinase revealed eight hit series, four of which are currently undergoing hit-to-lead chemistry.

### Supporting information

SMARTS definitions of unwanted groups (Table S1) and kinase core fragments (Table S2) are available free of charge on the internet. Python scripts can be obtained from the authors upon request.
